# Rapid protein profiling facilitates surveillance of invasive mosquito species

**DOI:** 10.1186/1756-3305-7-142

**Published:** 2014-03-31

**Authors:** Francis Schaffner, Christian Kaufmann, Valentin Pflüger, Alexander Mathis

**Affiliations:** 1 Swiss National Centre for Vector Entomology, Institute of Parasitology, University of Zurich, Winterthurerstrasse 266a, Zurich 8057, Switzerland; 2 Mabritec SA, Lörracherstrasse 50, Riehen 4125, Switzerland

**Keywords:** Invasive mosquitoes, Aedes, Surveillance, Monitoring, Mass spectrometry, Egg, Identification, Europe, North America

## Abstract

**Background:**

Invasive aedine mosquito species have become a major issue in many parts of the world as most of them are recognised vectors or potentially involved in transmission of pathogens. Surveillance of these mosquitoes (e.g. *Ae. aegypti*, Yellow fever mosquito, *Aedes albopictus*, Asian tiger mosquito) is mainly done by collecting eggs using ovitraps and by identification of the larvae hatched in the laboratory. In order to replace this challenging and laborious procedure, we have evaluated matrix-assisted laser desorption/ionization time of flight mass spectrometry (MALDI-TOF MS) for easy and rapid species identification.

**Methods:**

Individual protein profiles were generated using five eggs each of nine aedine species (*Ae. aegypti*, *Ae. albopictus*, *Ae. atropalpus*, *Ae. cretinus*, *Ae. geniculatus*, *Ae. japonicus*, *Ae. koreicus*, *Ae. phoeniciae*, *Ae. triseriatus*) from various geographical origins, and species-specific biomarker mass sets could be generated. A blinded validation using our reference data base for automated egg identification was performed. In addition, pools of 10 aedine eggs (132 two-species and 18 three-species pools) in different ratios were evaluated.

**Results:**

Specific biomarker mass sets comprising 18 marker masses could be generated for eggs of nine container-inhabiting aedine species, including all the major invasive and indigenous species of Europe and North America. Two additional masses shared by all investigated aedine species are used as internal calibrators. Identification of single eggs was highly accurate (100% specificity, 98.75% sensitivity), and this method is also of value for the identification of species in pools of ten eggs. When mixing two or three species, all were identified in all pools in at least 2 or 1 of the 4 loaded replicates, respectively, if the “lesser abundant” species in the pool accounted for three or more eggs.

**Conclusions:**

MALDI-TOF MS, which is widely applied for routine identification of microorganisms in clinical microbiology laboratories, is also suited for robust, low-cost and high throughput identification of mosquito vectors in surveillance programmes. This tool can further be developed to include a wide spectrum of arthropods but also other Metazoa for which surveillance is required, and might become the method of choice for their centralised identification via online platforms.

## Background

Invasive mosquitoes have become a major issue in many parts of the World [1,2], as most of them are potential vectors of arboviruses and parasites [3]. In Europe, the Asian tiger mosquito *Aedes albopictus* and the Asian bush mosquito *Ae. japonicus* are invading Southern and Central parts, respectively. The Yellow fever mosquito *Ae. aegypti*, with recently established populations in Madeira and along the Black Sea coast [4], is knocking at Europe’s doors, whereas other Asian and American mosquitoes such as *Ae. atropalpus* or *Ae. koreicus* locally occur at several places [4]. The danger posed by these mosquitoes in Europe was demonstrated by the very recent local transmissions of dengue and chikungunya viruses by *Ae. albopictus* in France and Croatia [5] and the outbreak of dengue in Madeira with *Ae. aegypti* acting as vector [6]. Statistical modelling revealed that more areas in Europe are climatically suitable for these two vector species [7]. In North America, the naturalised *Ae. aegypti* and the invasive *Ae. albopictus* mainly occur in south-eastern states, whereas *Ae. japonicus* has colonised most of the eastern states (except the southernmost ones) and some western states as well [8,9]. Also in North America, dengue fever is back [8], and the global context raises awareness for the emergence of chikungunya fever [10].

Thus, surveillance and control of invasive mosquitoes is essential to assess and manage the risks they induce [11]. Proactive surveillance is of particular relevance within an early-warning strategy, in order to detect populations of invasive mosquito species in time, before they are locally well-established and start to further spread. Suppressing such container-inhabiting invasive aedine mosquitoes in an urban environment is particularly challenging, mainly because of the diversity and limited accessibility of larval habitats, and their elimination has been achieved only in a context of early detection [5,12,13].

**Figure 1:**
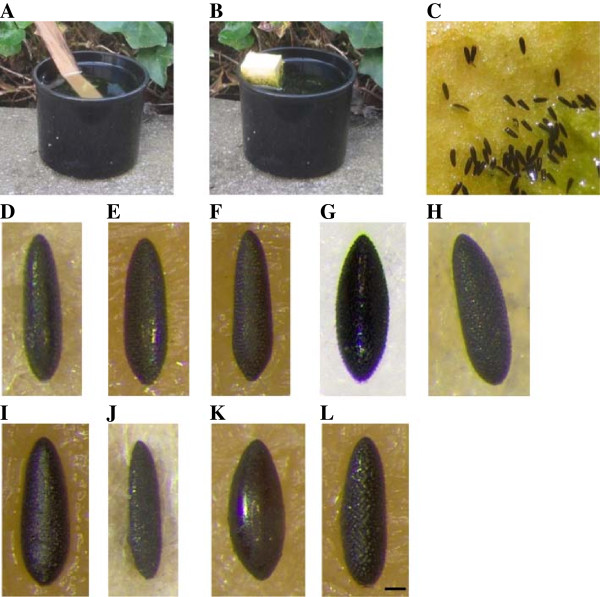
**Container-inhabiting aedine mosquito eggs collected with ‘ovitraps’ that are commonly used for monitoring purposes. (A)** Ovitrap with a wooden stick as oviposition support; **(B)** ovitrap with a floating piece of polystyrene as oviposition support; **(C)** eggs laid on polystyrene; eggs of **(D)***Ae. aegypti*, **(E)***Ae. albopictus*, **(F)***Ae. atropalpus*, **(G)***Ae. cretinus*, **(H)***Ae. geniculatus*, **(I)***Ae. japonicus*, **(J)***Ae. koreicus*, **(K)***Ae. phoeniciae*, **(L)***Ae. triseriatus*. Scale bar equates to 0.1 mm.

Surveillance of container-breeding mosquitoes is recommended to be performed using so called ‘ovitraps’ (Figure 1), which attract gravid females and thus provide presence and relative abundance data based on the eggs they deposit in the traps [4]. However, morphological identification of these eggs, mainly based on the observation of the exochorion ornamentation using reflecting (or episcopic) lightening, is time consuming, requires expertise, and not all species are precisely characterised [4]. Therefore, eggs are usually hatched to allow for larval identification [4], but hatching is often poor and is delayed in case of overwintering (diapausing) eggs. Genetic identification by PCR is described for only a few container-breeding aedine species [4,14], and this method is costly and laborious when extensive monitoring is desired. Matrix-assisted laser desorption/ionization time of flight mass spectrometry (MALDI-TOF MS), which has come of age for the high throughput, fast, accurate and low-cost identification of microorganisms in clinical diagnostic laboratories [15,16], has recently shown promise to identify metazoan organisms, including larval and adult stages of biting midges (Ceratopogonidae) and mosquitoes (Culicidae) [17-19]. This included the discriminatory identification of cryptic insect species [20,21], and the technique has proven its suitability for accurate identification of field-collected adult biting midges on a large scale [17]. Further, the method was useful to identify ageing biomarkers in *Ae. aegypti*[22].

Here, we developed MALDI-TOF MS for the identification of eggs of nine container-inhabiting aedine mosquito species that deposit their eggs in a comparable manner (*Ae. aegypti*, *Ae. albopictus*, *Ae. atropalpus*, *Ae. cretinus*, *Ae. geniculatus*, *Ae. japonicus*, *Ae. koreicus*, *Ae. phoeniciae*, *Ae. triseriatus*), including all the major invasive and indigenous species of Europe and North America.

## Methods

### Mosquito egg samples

Aedine eggs were either obtained from laboratory colonies or collected in the field using ovitraps with floating polystyrene blocks used as egg-laying devices (see Additional file 1: Table S1; Figure 1). From each batch of eggs collected in the field, at least 10 were hatched and reared to larvae and adults for morphological and genetic species confirmation [4,23]. In case that more than one species was obtained from a sample, only eggs from reared females were further used (F1). The remaining eggs were stored at 12°C ± 1°C under a short day regime (8/16 h L/D) and high humidity for further use (validation study).

### MALDI-TOF MS

#### Sample preparation

Single eggs were placed directly on a well of a slide, mixed with 1 μl formic acid (10%) and squeezed with forceps (Dumont Nr. 5). The egg suspension was then overlaid with 1 μl of a saturated solution of sinapic acid (saturated solution of sinapic acid in 60% acetonitrile, 40% H_2_O, 0.3% trifluoroacetic acid; Sigma-Aldrich, Buchs, Switzerland) and air-dried at room temperature. Pools of ten eggs were mixed with 10 μl formic acid (10%) in a 500 μl Multiply®-Pro or 200 μl Axygen® PCR tube and manually homogenised with a disposable pestle (Fisher Scientific, Wohlen, Switzerland). One μl of egg pool suspension were spotted in quadruplicate on a 48 well slide, overlaid with 1 μl of the saturated solution of sinapic acid and air-dried at room temperature.

#### Parameters

Protein mass fingerprints were obtained using a MALDI-TOF Mass Spectrometry Axima™ Confidence machine (Shimadzu-Biotech Corp., Kyoto, Japan), with detection in the linear, positive mode at a laser frequency of 50 Hz and within a mass range from 3,000-20,000 Da. Acceleration voltage was 20 kV, and the extraction delay time was 200 ns. A minimum of 10 laser shots per sample was used to generate each ion spectrum. For each insect sample, a total of 100 protein mass fingerprints were averaged and processed using the Launchpad™ v2.8 software (Shimadzu-Biotech Corp., Kyoto, Japan). Spectra were internally calibrated by the use of two conserved aedine egg masses (m/z 5660.1, m/z 11’321.8) with an error of 800 ppm in the Launchpad™ v2.8 software. This software was also used for peak processing of all raw spectra with the following settings: the advanced scenario was chosen from the Parent peak clean up menu, peak width was set 80 chans, smoothing filter width 50 chans, baseline filter width 500 chans and the threshold apex was chosen as peak detection method. For the threshold apex peak detection, the threshold type was set as dynamic, the threshold offset was set to 0.025 mV with a threshold response factor of 1.25. Each target plate was externally calibrated using the reference strain *Escherichia coli* DH5α.

#### Peak matrix generation for unsupervised cluster analysis

Generated protein mass fingerprints were analysed with SARAMIS™ Premium (spectral archive and microbial identification system, AnagnosTec, Potsdam-Golm, Germany). Binary matrix was generated using the SARAMIS™ SuperSpectra™ tool and exported to a text file. Intensity and error columns were removed with the Microsoft® Excel software. The adapted binary matrix was imported into the free software PAST v2.12. Using PAST, multivariate cluster analysis was performed using the paired group dice algorithm [24]. The generated dendrogram was exported in nexus file format and imported into the free FigTree v1.3.1 application for dendrogram illustration.

#### Superspectra generation

Generated protein mass fingerprints of 5 eggs each from 9 aedine mosquito species were analysed with SARAMIS™ Premium software, and biomarker mass patterns, called superspectra, were calculated for the 9 *Aedes* species using the SARAMIS™ SuperSpectra™ tool. To that end, the peak lists of all 45 *Aedes* eggs (reference set) were imported into the SARAMIS™ software, the spectra trimmed to a mass range of 3–20 kDa, and peaks with a relative intensity below 1% were removed. Peak lists were binned and average masses were calculated using the SARAMIS™ SuperSpectra™ tool with an error of 800 ppm. Specificities of these potential biomarker masses were determined by comparison against the whole SARAMIS™ spectral archive. In accordance with the SARAMIS user guidelines, the threshold for identification was set at 75% biomarker matches based on the reference data set. Twenty masses for each species were weighted and used as SuperSpectra™ for automated *Aedes* egg species identification (see Additional file 2: Table S2).

#### Superspectra validation

For SuperSpectra™ validation, 175 aedine single eggs and 150 egg pools in quadruplicates were analysed. The 775 generated mass fingerprints obtained were imported into SARAMIS™ software for automated identification with SuperSpectra™.

## Results and discussion

**Figure 2:**
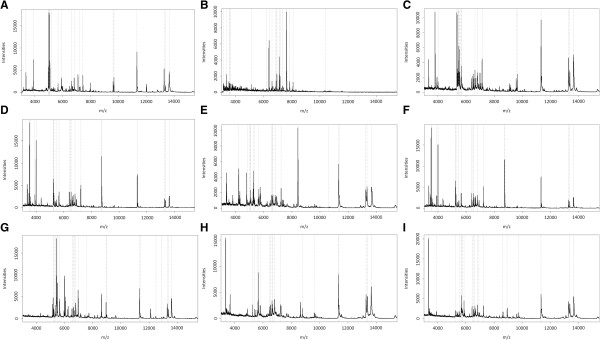
**Individual protein profiles of nine important aedine mosquitoes. (A)***Ae. aegypti*, **(B)***Ae. albopictus*, **(C)***Ae. atropalpus*, **(D)***Ae. cretinus*, **(E)***Ae. geniculatus*, **(F)***Ae. japonicus*, **(G)***Ae. koreicus*. **(H)***Ae. phoeniciae*, **(I)***Ae. triseriatus*. Biomarker masses are illustrated as dashed lines within the range of 3–30 kDa.

**Figure 3:**
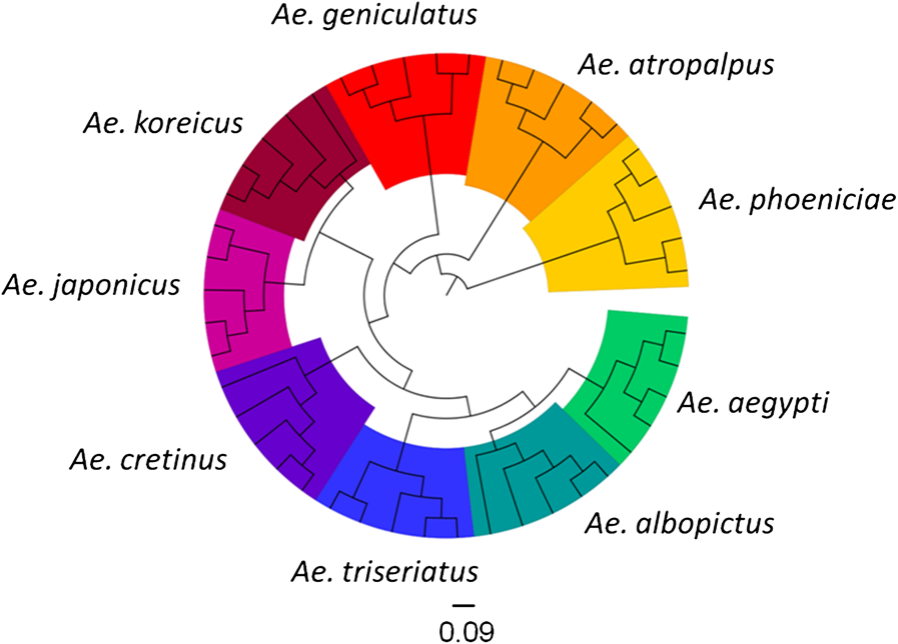
**Dendrogram of matrix-assisted laser desorption/ionization time of flight (MALDI-TOF) mass spectra of five single eggs for each of the nine studied aedine species.** Distance units correspond to the relative similarity calculated from the distance matrix.

In a first step, individual protein profiles (Figure 2) were generated using five eggs of each mosquito species from various geographical origins (Additional file 1: Table S1). These entire protein profiles (data count between 78 and 157) were used to compile the total mass spectra for the nine species in a dendrogram, yielding distinct clustering of the same species on definite branches (Figure 3). Species-specific biomarker mass sets of 18 marker masses could be generated (Figure 2; Additional file 2: Table S2) and were imported into the SARAMIS™ software, adding to the >3400 biomarker mass sets, including 54 species-specific insect (larvae and adults) sets, of our reference data base (http://www.mabritec.com). In addition, two masses were identified that are shared by all investigated aedine species (see Additional file 2: Table S2), and these masses were henceforth used as internal calibrators.

A blinded validation using our reference database for automated egg identification was performed. Out of 175 single eggs from all the nine container-breeding invasive or indigenous aedine species included in the study, 172 were accurately identified (specificity 100%, overall sensitivity 98.3%, Table 1); the remaining three eggs (1.7%) yielded poor protein profiles, probably because they were unfertilised or desiccated.

**Table 1:** **Results of a blinded validation for automated MALDI**-**TOF MS identification of single eggs using our reference data base**

**Species**	**No. identified eggs**	**No. unidentified eggs**	**Sensitivity %**
*Aedes albopictus*	18	1	94.7
*Ae. aegypti*	19	0	100
*Ae. atropalpus*	14	0	100
*Ae. cretinus*	7	0	100
*Ae. geniculatus*	55	1	98.2
*Ae. japonicus*	23	1	95.8
*Ae. koreicus*	7	0	100
*Ae. phoeniciae*	7	0	100
*Ae. triseriatus*	22	0	100

In a second step, we aimed at improving the performance and reducing the costs of MALDI-TOF MS applied in surveillance programmes by identifying mosquito species in batches of eggs. First, 132 two-species pools containing ten aedine eggs in different ratios from the three container-inhabiting aedine species occurring in central Europe (*Ae. albopictus*, *Ae. geniculatus*, *Ae. japonicus*) and from the yellow fever mosquito *Ae. aegypti* were analysed with 4 technical replicates per pool. At least one species could be identified in every single replicate. The more balanced the composition of the pools was, the more replicates provided identification of both species (Table 2): Thus, both species were identified in all pools in at least 2 of the 4 replicates if the “lesser abundant” species in the pool accounted for three or more eggs. Single replicates of the pools yielded the identification of both species in 25% of the pools in the extreme ratio of 9 and 1 eggs.

**Table 2:** **Sensitivity of egg identification in two**-**species pools of ten eggs in different mix ratios**

**Mix ratio**	**10/0**	**9/1**	**8/2**	**7/3**	**6/4**	**5/5**
Number of pools	24	24	24	24	24	12
Total number of spots measured (technical replicates)	96	96	96	96	96	48
Number of the 4 technical replicates yielding identification of both species						
4	0.0%	0.0%	0.0%	70.3%	75.0%	91.7%
≥3	0.0%	0.0%	16.7%	91.7%	91.7%	100.0%
≥2	0.0%	12.5%	20.8%	100.0%	100.0%	100.0%
≥1	0.0%	25.0%	37.5%	100.0%	100.0%	100.0%

Three biological replicates have been performed per pools containing *Ae. aegypti*/*Ae. japonicus*, *Ae. albopictus*/*Ae. geniculatus*, *Ae. albopictus*/*Ae. japonicus*, *Ae. geniculatus*/*Ae. japonicus*.

We experienced an improved reproducibility of identification when calibration of the spectra was done with the mosquito-derived internal calibrator masses, as compared with calibration relying on the external calibrators (*Escherichia coli*). For example, the sensitivity for the identification of two-species pools improved from 84.5% to 91.7% in the case of the 5:5 egg pools.

Then, 18 three-species pools (i.e. *Ae. aegypti*, *Ae. albopictus*, *Ae. japonicus*) of ten eggs were evaluated. Based on the experience with the two-species pools (reliable identification of eggs constituting at least 30%), pools containing 3, 3, 4 eggs in all permutations were tested. Again, at least one species could be identified in every single replicate, and at least one technical replicate per pool was positive for all three species in 97.6% (Table 3).

**Table 3:** **Sensitivity of egg identification in three**-**species pools of ten eggs in different mix ratios**

**Mix ratios**	**3/3/4**
Number of pools tested	18
Total number of spots measured (technical replicates)	72
Number of the 4 technical replicates yielding identification of 2 species	
4	95.80%
≥3	100.00%
≥2	100.00%
≥1	100.00%
Number of the 4 technical replicates yielding identification of all 3 species	
4	87.50%
≥3	88.80%
≥2	94.40%
≥1	97.60%

Six biological replicates have been performed per pools containing *Ae. aegypti*/*Ae. albopictus*/*Ae. japonicus* in ratio 3/3/4 in all three possible variations.

## Conclusions

Thus, accurate identification by MALDI-TOF MS is possible for mosquito eggs, also in pools, at least for species which account for 30% or more of the eggs. This is particularly valuable in situations where detection of a low abundant species is not necessary (i.e. during a pathogen transmission period). Further, if experienced personnel are available, the sensitivity of egg identification in pools could be improved by preselecting eggs of similar shape (Figure 1).

Further developments will aim at investigating larger pools of mosquito eggs and expanding the database to also include the remaining mosquito species whose eggs can be encountered in surveillance programmes of container-inhabiting mosquitoes in Europe and North America, i.e. species usually breeding in tree holes or rock pools in southern Europe (*Ae. berlandi*, *Ae. echinus*, *Ae. gilcolladoi*, *Ae. mariae*, *Ae. pulcritarsis*, *Ae. zammitii*, and *Orthopodomyia pulcripalpis*) or North America (*Ae. hendersoni*, *Ae. thibaulti*, *Ae. togoi*, *Ae. varipalpus*, *Ae. zoosophus*, *Or. alba*, *Or. signifera*).

Taken together, we showed that protein profiling, which is a quick tool with low operational costs, is reliable and accurate for species identification of eggs of invasive/indigenous aedine mosquito species. This approach has successfully been pursued during a recent surveillance programme of *Ae. albopictus* in Switzerland [25], revealing its presence at seven of the 30 sampled sites. Three of these positive sites were located north of the Alpine crest, where the species was not known to occur, in an area largely occupied by the indigenous *Ae. geniculatus* and the invasive *Ae. japonicus*, which were identified at two of them as well. This demonstrates the usefulness of the described method in an applied context. Further, protein profiling of eggs would also identify invasive mosquito species not expected in a monitored area and which therefore might not be considered in DNA-based approaches.

The simple and rapid preanalytical procedure for protein profiling can be done in peripheral laboratories and the slides sent to the measuring laboratory. The future application of the method will include the accomplishment of the measurement with a mass spectrometry device anywhere and the identification via our online platform.

## Competing interests

VP is employed by Mabritec SA, a commercial service laboratory. The other authors declare that they have no competing interests.

## Authors’ contributions

All authors designed the research; FS collected the samples; CK and VP performed research and analysed data. All authors contributed to the writing of the manuscript and have approved the final version.

## Supplementary Material

Additional file 1: Table S1 Origin and suppliers of aedine egg samples. Click here for file

Additional file 2: Table S2 Biomarker marker masses used for the identification of eggs of nine aedine mosquito species. Grey cells: conserved masses used for internal calibration (unit: m/z). Click here for file
