# Supplementary material for: Rapid protein profiling facilitates surveillance of invasive mosquito species
Source: Parasit Vectors. 2014 Mar 31;7:142. doi: 10.1186/1756-3305-7-142 (PMC4022357; doi:10.1186/1756-3305-7-142)
Supplement: Additional file 1: Table S1 — Origin and suppliers of aedine egg samples. [file 1756-3305-7-142-S1.pdf]

Additional file 1 - Origin and suppliers of aedine egg samples.

Species	Strain/field samples[1]	Origin	Provided by
Ae. aegypti	UGAL strain	Georgia, USA	Prof. M.R. Brown, UGA, USA
	Manatee County (1)	Florida, USA	Prof. D. Fonseca, CVB-Rutgers, USA
	Rock (2)*	Cuba	Dr. P. Müller, Swiss TPH, Switzerland
	Field sample (2)	New Caledonia	Dr. L. Guillaumot, IPNC, New Caledonia
Ae. albopictus	Liguria 2011 (1)*	Liguria, Italy	Dr. C. Kaufmann, IPZ, Switzerland
	Monmouth County (2)	New Jersey, USA	Prof. D. Fonseca, CVB-Rutgers, USA
	St. Johns County (1)	Florida, USA	Prof. D. Fonseca, CVB-Rutgers, USA
	ATM-HI10 (1)	Hawaii, USA	Prof. D. Fonseca, CVB-Rutgers, USA
	MRA-804 (CDC)	Florida, USA	Prof. M.R. Brown, UGA, USA
	Field samples	Albania	Dr. E. Dikolli, IPH, Albania
Ae. atropalpus	Bass Rock strain (2)	Arizona, USA	Prof. M.R. Brown, UGA, USA
	RPM-NJ95 (3)	New Jersey, USA	Prof. D. Fonseca, CVB-Rutgers, USA
	Tallulah 2007	Georgia, USA	Prof. G. O'Meara, FMEL, Vero Beach, USA
Ae. cretinus	Lab strain Athens (5)	Attica, Greece	Dr. G. Koliopoulos, BPI, Greece
Ae. geniculatus	Field samples (2)	Alsace, France	Dr. F. Schaffner, IPZ, Switzerland
	Zurich 2012*	Zurich, Switzerland	Dr. C. Kaufmann, IPZ, Switzerland
	Field samples	Vaud, Switzerland	Dr. O. Glaizot, UNIL, Switzerland
	Field samples (3)	Namur, Belgium	Prof. M. Coosemans, ITM, Belgium
Ae. j. japonicus	Field samples (1)	Zurich, Switzerland	Dr. C. Kaufmann, IPZ, Switzerland
	Field samples (1)	Uri, Switzerland	C. Wüthrich, AFU, Switzerland
	ARPM-PA07 (2)*	Pennsylvania, USA	Prof. D. Fonseca, CVB-Rutgers, USA
	ARPM-HI10 (1)	Hawaii, USA	Prof. D. Fonseca, CVB-Rutgers, USA
Ae. koreicus	Field samples (5)	Limburg, Belgium	Dr. F. Schaffner, IPZ, Switzerland
Ae. phoeniciae	Field samples (5)	Paphos, Cyprus	Dr. F. Schaffner, IPZ, Switzerland
Ae. triseriatus	ETM-NJ95 (3)	New Jersey, USA	Prof. D. Fonseca, CVB-Rutgers, USA
	FMEL colony (2)	Florida, USA	Prof. P. Lounibos, FMEL, Vero Beach, USA

[1]In brackets: numbers of eggs used to develop the SuperSpectra™. *Origin of eggs used in the experiments with pooled eggs.
