# Supplementary material for: Rapid protein profiling facilitates surveillance of invasive mosquito species
Source: Parasit Vectors. 2014 Mar 31;7:142. doi: 10.1186/1756-3305-7-142 (PMC4022357; doi:10.1186/1756-3305-7-142)
Supplement: Additional file 2: Table S2 — Biomarker marker masses used for the identification of eggs of nine aedine mosquito species. Grey cells: conserved masses used for internal calibration (unit: m/z). [file 1756-3305-7-142-S2.pdf]

Additional file 2 - Biomarker marker masses used for the identification of eggs of nine aedine mosquito species.

Grey cells: conserved masses used for internal calibration (unit: m/z).

Aedes aegypti	*Aedes albopictus*	*Aedes atropalpus*	*Aedes cretinus*	*Aedes geniculatus*	*Aedes japonicus*	*Aedes koreicus*	*Aedes phoeniciae*	*Aedes triseriatus*
3'365.9	3'365.9	5'201.9	3'528.6	3'369.7	5'341.8	3'807.7	3'159.9	3'365.9
3'923.7	3'678.7	5'259.9	3'586.2	4'796.3	5'357.3	4'026.8	3'369.7	3'906.4
5'277.5	5'259.9	5'341.8	3'923.7	4'854.1	5'382.3	5'357.3	3'586.2	4'966.5
5'542.4	5'417.9	5'476.1	4'010.1	5'047.0	5'417.9	5'399.1	3'624.1	5'009.9
5'660.1	5'615.2	5'660.1	5'250.8	5'087.7	5'526.6	5'453.7	3'638.2	5'026.9
5'743.8	5'660.1	6'540.4	5'277.5	5'259.9	5'542.4	5'476.1	5'129.4	5'067.5
5'872.0	5'803.0	6'555.4	5'291.9	5'291.9	5'603.4	5'506.7	5'660.1	5'087.7
6'438.2	6'226.6	6'684.3	5'439.8	5'341.8	5'660.1	5'542.4	6'226.6	5'183.4
6'535.5	6'464.6	6'796.7	5'660.1	5'660.1	5'702.6	5'660.1	6'392.8	5'660.1
6'638.9	6'535.5	6'970.1	6'403.8	6'555.4	6'392.8	6'638.9	6'604.0	5'916.7
6'817.5	6'638.9	7'537.6	6'488.3	6'624.0	6'624.0	6'817.5	6'868.4	6'638.9
6'928.3	6'817.5	8'957.2	6'535.5	6'836.4	6'664.0	7'144.4	6'887.0	7'202.6
7'239.4	7'239.4	9'001.7	6'817.5	7'239.4	6'817.5	7'447.8	6'944.6	7'447.8
8'625.5	8'761.3	11'321.8	7'239.4	7'331.5	8'668.5	8'925.1	7'113.8	8'668.5
8'925.1	9'618.0	11'364.7	8'740.4	7'405.4	9'190.7	9'554.5	7'170.6	9'618.0
9'001.7	11'321.8	12'483.8	8'761.3	8'443.6	11'321.8	9'636.6	7'629.3	9'679.0
9'706.2	11'364.7	12'888.8	11'321.8	11'321.8	11'364.7	11'321.8	7'645.0	11'321.8
11'321.8	13'277.2	13'298.1	13'277.2	13'277.2	13'246.6	13'277.2	7'847.5	13'277.2
13'357.0	13'357.0	13'378.6	13'357.0	13'357.0	13'327.0	13'357.0	8'088.4	13'357.0
13'634.7	13'634.7	13'594.7	13'634.7	13'674.4	13'634.7	13'634.7	10'398.1	13'634.7
